# Immediate Carotid Artery Stenting or Deferred Treatment in Patients With Tandem Carotid Lesions Treated Endovascularly for Acute Ischaemic Stroke

**DOI:** 10.1016/j.ejvsvf.2023.12.002

**Published:** 2023-12-19

**Authors:** Theodora van Elk, Louise Maes, Anne van der Meij, Robin Lemmens, Maarten Uyttenboogaart, Gert J. de Borst, Clark J. Zeebregts, Paul J. Nederkoorn

**Affiliations:** a Department of Neurology, University Medical Centre Groningen, Groningen, the Netherlands; b Department of Neurology, University Hospital Leuven, Leuven, Belgium; c Department of Neurosciences, Experimental Neurology, KULeuven - University of Leuven, Leuven, Belgium; d Department of Neurology, Amsterdam University Medical Centre, Amsterdam, the Netherlands; e Department of Neurology, Leiden University Medical Centre, Leiden, the Netherlands; f Department of Surgery (Division of Vascular Surgery), University Medical Centre Utrecht, Utrecht, the Netherlands; g Department of Surgery (Division of Vascular Surgery), University Medical Centre Groningen, Groningen, the Netherlands

**Keywords:** Acute ischaemic stroke, Carotid artery, Carotid artery stenting, Endovascular thrombectomy, Large vessel occlusion, Tandem lesion

## Abstract

Fifteen to 20% of patients with an acute ischaemic stroke have a tandem lesion defined by the combination of an intracranial large vessel thrombo-embolic occlusion and a high grade stenosis or occlusion of the ipsilateral internal carotid artery. These patients tend to have worse outcomes than patients with isolated intracranial occlusions, with higher rates of disability and death. The introduction of endovascular thrombectomy to treat the intracranial lesion clearly improved the outcome compared with treatment with intravenous thrombolysis alone. However, the best treatment strategy for managing the extracranial carotid artery lesion in patients with tandem lesions remains unknown. Current guidelines recommend carotid endarterectomy for patients with transient ischaemic attack or non-disabling stroke and moderate or severe stenosis of the internal carotid artery, within two weeks of the initial event, to prevent major stroke recurrence and death. Alternatively, the symptomatic carotid artery could be treated by endovascular placement of a stent during endovascular thrombectomy (EVT). This would negate the need for a second procedure, immediately reduce the risk of stroke recurrence, increase patient satisfaction, and could be cost effective. However, the administration of dual antiplatelet therapy could potentially increase the risk of symptomatic intracranial haemorrhage in patients with acute ischaemic stroke. Randomised controlled trials evaluating the efficacy and safety of immediate carotid artery stenting during EVT in acute stroke patients with tandem lesions are currently ongoing and will impact the current guidelines regarding the treatment of patients with acute ischaemic stroke due to these tandem lesions.

## Highlights

• Tandem lesions occur in approximately 15–20% of patients with an acute ischaemic stroke.
• These patients have higher rates of disability and death compared to patients with isolated intracranial occlusions.
• The best treatment strategy for the extracranial internal carotid artery lesion remains unknown.
• Carotid artery stenting during EVT could immediately reduce the risk of recurrent stroke.
• RCT’s evaluating the efficacy and safety of CAS during EVT in tandem lesion patients are currently ongoing.

## Introduction

Approximately 24–46% of acute ischaemic strokes are caused by occlusion of a large proximal intracranial cerebral artery, a so called large vessel occlusion (LVO).^1^ Patients with LVOs usually have a large area of hypoperfusion, resulting in severe neurological deficits. LVOs can develop through various mechanisms: (1) embolism resulting from atherosclerotic plaque rupture in extracranial arteries leading to occlusion of an intracranial vessel (artery to artery embolism), (2) cardio-embolic events related to cardiac disease such as atrial fibrillation, (3) intracranial atherosclerosis with secondary thrombosis, and (4) other causes such as dissection or cryptogenic causes of vessel occlusion.^2^ The presence of an LVO is associated with 4.5 fold increased odds of death (95% confidence interval [CI] 2.7–7.3) and a threefold reduction in the odds of good outcome compared with patients without an LVO.^3^

## Acute re-perfusion treatments

In 1995, the field of acute ischaemic stroke treatment was revolutionised with the introduction of intravenous tissue plasminogen activator (IV-tPA), which dissolves the thrombus.^4^ IV-tPA remained the primary treatment paradigm for approximately two decades until 2015, when multiple randomised clinical trials (RCTs)^5^, ^6^, ^7^, ^8^, ^9^ demonstrated the efficacy and safety of endovascular thrombectomy (EVT) for the treatment of LVOs. EVT is an endovascular procedure in which the clot causing hypoperfusion is mechanically removed through aspiration or stent retrieval. A pooled meta-analysis of these five RCTs demonstrated that EVT more than doubles the odds of good functional outcome compared with standard therapy alone, without any significant difference in death or risk of haemorrhage at 90 days.^10^ The number needed to treat to improve the degree of disability measured with the modified Rankin scale (mRS) score in one patient by at least one point is 2.6.^10^

Since then, additional clinical trials have shown the efficacy of EVT in addition to standard medical care for patients with acute ischaemic stroke due to LVO, leading to improved outcomes when EVT is performed within either six hours,^5^^,^^8^^,^^9^^,^^11^^,^^12^ eight hours,^7^ or 12 hours^6^ of symptom onset. Thereafter, clinical trials have demonstrated that the time window could be further extended to up to 24 hours after symptom onset in patients with favourable mismatch patterns: either a mismatch between infarct size and clinical deficit, perfusion lesion (suggestive of a large penumbra), or presence of collateral flow observed on computed tomography angiography.^13^, ^14^, ^15^ Even patients presenting with large core volumes can still benefit although, generally, these patients have worse outcomes than patients with smaller ischaemic infarcts at baseline.^16^ The subsequent expansion of the EVT treatment time window has drastically improved the prognosis for patients with LVOs.

## Tandem lesions

**Figure 1:**
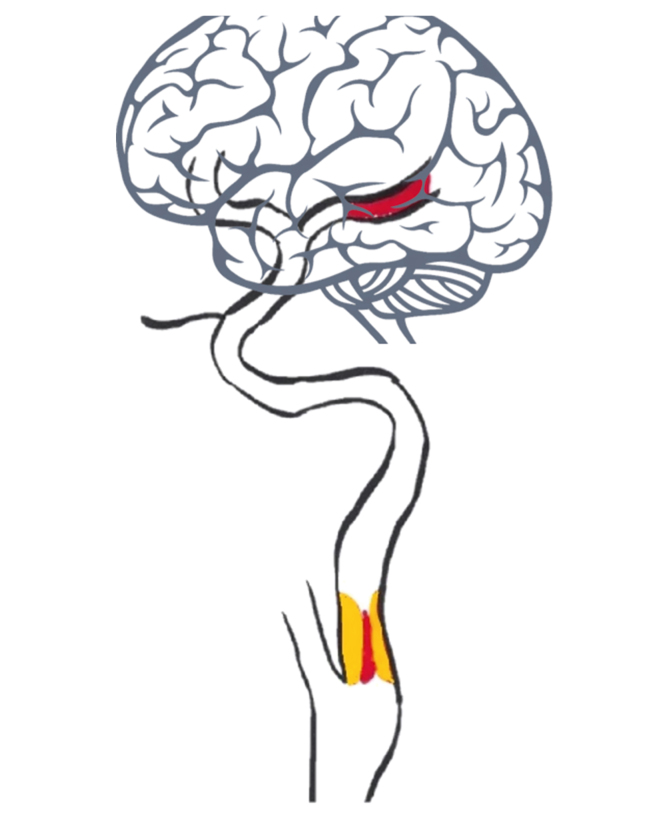
Diagram of tandem lesion with a large vessel occlusion and an ipsilateral carotid artery stenosis of atherosclerotic origin.

Tandem lesions are here defined as the combination of an intracranial LVO and a concomitant stenosis >50% or occlusion in the ipsilateral extracranial carotid artery, depicted in Figure. 1. These lesions occur in approximately 15–20% of patients experiencing an acute ischaemic stroke. Tandem lesions are most often caused by atherosclerosis, although they can also result from arterial dissection, carotid web, or the presence of a floating thrombus.

Patients with tandem lesions have higher rates of disability and death than patients with isolated intracranial occlusions. Treatment with IV-tPA alone results in favourable outcomes in only 30% of patients, with mortality rates up to 50%.^17^ Low recanalisation rates are most likely due to increased clot burden and the stenosis creating low flow which impedes the delivery of thrombolytic agents to the intracranial thrombus.^18^ However, despite these limitations, endovascular treatment of tandem lesions clearly improves outcomes compared with treatment with intravenous thrombolysis (IVT) alone. This was demonstrated in the HERMES collaboration meta-analysis, where the treatment effect of EVT in patients with tandem lesions was similar to that of patients with isolated intracranial occlusions, resulting in a favourable functional outcome in 46% of patients.^10^

Nevertheless, the best treatment strategy for managing the extracranial atherosclerotic carotid artery lesion in patients with a tandem lesion remains unknown. Current guidelines recommend carotid endarterectomy (CEA) or carotid artery stenting (CAS) for patients with stroke and moderate or severe stenosis of the internal carotid artery within two weeks of the initial event to prevent stroke recurrence.^19^^,^^20^ For disabling stroke, best medical management alone is often chosen while forgoing CEA or CAS. However, the introduction of EVT has enabled the simultaneous treatment of the carotid artery stenosis during the EVT procedure, thus avoiding a deferred secondary invasive procedure. The safety and efficacy of this treatment strategy, CAS during EVT, is currently a matter of debate.

## Carotid artery stenting

CAS during EVT improves cerebral blood flow and has the potential to immediately reduce the risk of recurrent stroke. Some interventional radiologists even claim that stenting is necessary to perform a successful intracranial thrombectomy.^21^ Compared with CEA, CAS is less invasive and could increase patient satisfaction. CAS during EVT could potentially decrease healthcare costs by avoiding a second procedure. However, in the acute setting it is difficult to predict how a patient will recover from the index event. Consequently, a proportion of patients who do not recover well, may have received futile stenting as typically no CEA or CAS would be considered in patients with remaining severe disabling symptoms.

The optimal timing of stenting during EVT remains unclear. The carotid artery lesion can be treated before the intracranial thrombectomy, i.e., the antegrade approach, improving access to the intracerebral occlusion, but delaying intracranial recanalisation. A possible disadvantage of the antegrade approach is the potential snagging of a retrievable stent in the already deployed carotid stent. Treating the intracranial occlusion first, i.e., the retrograde approach, allows for the rapid restoration of perfusion to the affected territory. This potentially increases the likelihood of a favourable outcome.^22^^,^^23^ However, this approach carries the risk of distal embolisation following initial successful re-perfusion. Another possible complication during the procedure is the development of bradycardia and hypotension due to activation of the baroreceptors in the carotid artery wall, which might increase final infarct core.

Immediate CAS during EVT necessitates the initiation of dual antiplatelet treatment to prevent in stent thrombosis in the months following stent placement. The need for dual antiplatelet therapy is often stated as an argument to avoid immediate CAS because of the feared risk of secondary haemorrhagic transformation of the recently infarcted brain tissue. Additionally, the rare complication of cerebral hyperperfusion syndrome could lead to an increased risk of intracranial haemorrhage.^24^ The rate of intracranial haemorrhage varies greatly between retrospective studies: some suggest higher rates,^25^ others, including the STRATIS and TITAN registries, suggest no excess risk of bleeding in patients treated with immediate CAS during EVT.^26^, ^27^, ^28^ Consensus regarding the antithrombotic management for carotid stent placement in the setting of endovascular stroke treatment is lacking, leading to variation in antithrombotic regimens.

Despite these concerns, systematic reviews, retrospective case series and patient registries have shown an association between performing CAS during EVT and successful re-perfusion as well as improved 90 day functional outcome, without differences in mortality rate,^18^^,^^22^^,^^26^^,^^29^, ^30^, ^31^, ^32^, ^33^, ^34^, ^35^ as shown in Table 1. The absence of data from robust RCTs hampers firm recommendations about optimal management, as reflected in the recent European Society of Vascular Surgery guideline.^36^

**Table 1:** Summary of registry articles and systematic reviews outlining functional outcomes, rates of successful re-perfusion, incidence of symptomatic intracranial haemorrhage and all cause mortality rates when endovascular thrombectomy (EVT) is combined with carotid artery stenting (CAS).

*Article*	Registry	Good outcome (mRS 0–2)	Successful reperfusion (mTICI 2b–3)	Symptomatic intracranial haemorrhage	All cause mortality
*CAS vs. no CAS*					
*Sivan-Hoffman et al.*^30^	11 studies *n* = 237	44% (33–55) (10 studies)	81% (73–89)	7% (2–13) (8 studies)	13% (8–20) (10 studies)
*Sadeh-Gonik et al.*^31^	13 studies *n* = 590	50% (42–59)	75% (69–81)	8% (6–11)	16% (11–22)
*Gory et al.*^32^	*n* = 395	52.2% (47.2–57.1)	76.7% (72.5–80.9)	5.3% (3.1–7.6)	13.2% (9.8–16.6)
*Jadhav et al.*^26^	*n* = 147	68.5% *vs.* 42.2% (*p* = .003)	87.0% *vs.* 83.7% (*p* = .79)	2.9 *vs.* 0% (*p* = .51)	12.3% *vs.* 10.9% (*p* = 1.0)
*Dufort et al.*^18^	21 studies *n* = 1635	OR 1.43 (1.07–1.91)		OR 1.41 (0.91–2.19)	OR 0.80 (0.50–1.28)
*Anadani et al.*^34^	Pooled analysis TITAN and ETIS *n* = 603 (CAS *n* = 341)	aOR 1.09 (1.01–1.19)	aOR 1.19 (1.11–1.27)	aOR 1.05 (0.99–1.09)	aOR 0.99 (0.93–1.04)
*Collette et al.*^35^	*n* = 344 (CAS *n* = 169) MR CLEAN registry	OR 1.32 (0.88–1.98)	OR 0.91 (0.60–1.36)	OR 0.61 (0.26–1.41)	
*CAS vs. balloon angioplasty*					
*Hellegering et al.*^29^	21 studies *n* = 1758	OR 1.99 (1.35–2.93)		OR 0.48 (0.16–1.45)	OR 1.27 (0.73–2.19)
*Zevallos et al.*^22^	34 studies *n* = 3 911	OR 1.95 (1.24–3.05)	OR 1.89 (1.26–2.83)	OR 1.31 (0.62–1.27)	OR 0.79 (0.50–1.27)
*IVT vs. no IVT*					
*Anadani et al.*^33^	TITAN registry *n* = 205	62% *vs.* 51% (*p* = .15)	82% *vs.* 80% (*p* = .86)	5% *vs.* 8% (*p* = .54)	8% *vs.* 20% (*p* = .017)

Data are presented as %, OR, or aOR with 95% CI as indicated in the table.

CI = confidence interval; EVT = endovascular thrombectomy; CAS = carotid artery stenting; aOR = adjusted odds ratio; mRS = modified Rankin scale; mTICI = modified Thrombolysis in Cerebral Infarction.

## Trials Of Carotid Artery Stenting During Endovascular Thrombectomy

There are currently four ongoing prospective randomised trials: the EASI-TOC trial in Canada (NCT04261478), the Thrombectomy in Tandem lesion (TITAN) trial (NCT03978988) in France, the Proximal Internal Carotid Artery Acute Stroke Secondary to Tandem or Local Occlusion Thrombectomy Trial (PICASSO) trial (NCT05611242) in the United States, and the Carotid Artery Stenting during Endovascular treatment of acute ischaemic Stroke (CASES) trial in The Netherlands and Belgium (ISRCTN14956654).

## Cases trial

CASES is a phase 3 international multicentre RCT with open label treatment and blinded outcome assessment (PROBE design) and a non-inferiority design. This clinical trial will be performed among thrombectomy capable stroke centres in The Netherlands and Belgium and is funded by governmental funding agencies (ZonMW/KCE). Patients with a computed tomography angiogram proven intracranial LVO in the anterior circulation and a symptomatic proximal ipsilateral carotid artery stenosis (>50%) or occlusion of presumed atherosclerotic origin will be randomised to either immediate CAS during EVT or to a deferred treatment strategy according to the current guidelines (CEA, CAS, or best medical management) and depending on the functional recovery of the patient. The primary endpoint is functional outcome at 90 days, assessed by the ordinal score on the mRS. Secondary outcomes include excellent functional outcome (mRS 0–1), good functional outcome (mRS 0–2), stroke severity measured with the National Institutes of Health Stroke Scale at 24 hours and at five to seven days, infarct volume at 24 hours, recurrence of ischaemic events, carotid re-occlusion, symptomatic intracranial haemorrhage, mortality rate, and the quality of life at 90 days. By evaluating efficacy and safety of immediate CAS during EVT of patients with acute stroke with tandem lesions, it is hoped to demonstrate non-inferiority of the immediate CAS approach, which would impact current guidelines regarding treatment recommendations in patients with acute ischaemic stroke due to tandem lesions. See http://www.cases-trial.eu/ for more information on the CASES trial.

## Conflicts of interest

None.

## Funding

None.
